# Patient Experience with Their Health Care Provider Among Non-Pregnant Women of Childbearing Age with Diabetes Mellitus by Race and Ethnicity in the United States

**DOI:** 10.1089/whr.2022.0037

**Published:** 2023-01-24

**Authors:** Kyrah K. Brown, Tiffany B. Kindratt, Grace Ellen Brannon, Bala Yadu Vamsi Sankuratri, Godfred O. Boateng

**Affiliations:** ^1^ Department of Kinesiology, University of Texas at Arlington, Arlington, Texas, USA.; ^2^ Department of Communication, University of Texas at Arlington, Arlington, Texas, USA.

**Keywords:** diabetes mellitus, health disparities, Medical Expenditure Panel Survey, ethnicity, race concordance, maternal health

## Abstract

**Objectives:**

The study objective was to investigate differences in patient experiences with health care providers among non-pregnant women of childbearing age with diabetes mellitus (DM) by race/ethnicity.

**Design:**

This study used cross-sectional data from the 2012–2018 Medical Expenditure Panel Survey. The sample was limited to women of childbearing age (18–45 years) who have ever been told they had diabetes (*n* = 763; weighted *n* = 903,670). The key independent variable was race/ethnicity. The variables of interest included patient experiences with health care in the past 12 months: patient-provider communication (PPC); patient-provider racial/ethnic concordance; patient-provider gender concordance; and satisfaction.

**Results:**

After adjusting for age, marital status, education, poverty level, health insurance, and perceived health status, non-Hispanic (NH) Black women had lower odds (adjusted odds ratio [aOR] = 0.04; 95% confidence interval [CI] = 0.01–0.11) of receiving care from a health care provider of the same race compared with NH white women. Similar results were found among Hispanic and NH women of other or multiple races. Hispanic women had lower odds (aOR = 0.18; 95% CI = 0.06–0.50) of seeing a health care provider of the same race/ethnicity compared with NH white women in adjusted models. There were no statistically significant differences in PPC, patient-provider gender concordance, and satisfaction with their health care provider among Hispanic, NH Black, or NH women of other or multiple races in comparison to NH White women.

**Conclusion:**

There is a need to improve PPC quality and satisfaction in this patient population. Patient-provider racial/ethnic discordance among women of color with DM is concerning given the existing diabetes-related disparities. More research on women with DM is needed to inform and improve patient experience and health outcomes.

## Introduction

Diabetes mellitus (DM) is a group of metabolic diseases characterized by inappropriately elevated blood glucose levels and inadequate production of insulin.^1^ In the United States (U.S.), 4.5% of non-pregnant women of childbearing age (18–49 years) have been diagnosed with DM.^2^ It is estimated that 30% of non-pregnant women of childbearing age living with DM are undiagnosed. Also, among non-pregnant women of childbearing age diagnosed with DM, the prevalence of uncontrolled diabetes is 51.5%.

Overall, the prevalence of DM among non-pregnant women of childbearing age is steadily increasing.^3–6^ Persistent racial and ethnic disparities in DM diagnosis and control exist in the U.S. population. The prevalence of diagnosed and uncontrolled DM is highest among non-Hispanic (NH) Black women, followed by Hispanic women, NH White women, and NH Asian women.^6,7^ Considering that nearly half of all U.S. pregnancies are unplanned, women entering pregnancy with DM, especially uncontrolled or undiagnosed DM, are at increased risk of long-term health complications^1^ and adverse maternal and infant health outcomes.^8^ Therefore, the American Diabetes Association (ADA) recommends targeted diabetes care and education in this population before potential pregnancy.^8^

Despite the documented need to optimize diabetes care and education among non-pregnant women of childbearing age at risk of diabetes, quality of health care experiences in this population has not been systematically investigated.^7,9^ An examination of the patient experiences of non-pregnant women of childbearing age with DM may provide insights into potential clinical and organizational interventions designed to improve DM outcomes in this population.

Patient experience, an important indicator of diabetes care quality, “encompasses the range of interactions that patients have with the health care system, including their care from health plans and from doctors, nurses, and staff in hospitals and physician practices.”^10^ Positive patient experience is associated with self-rated health outcomes and adherence to recommended medication and treatment.^11^ Positive patient experience is also associated with clinical effectiveness and patient safety, which suggests that patients' subjective experiences are valuable in examining health care encounters.^11^

On the other hand, negative patient experiences contribute to adverse outcomes such as medication non-adherence, which contributes to ∼125,000 preventable deaths in the U.S.^12^ An understanding of the patient experiences of racially and ethnically diverse non-pregnant, women of childbearing age with DM can aid in identifying clinical and policy interventions designed to improve patient-provider interactions and diabetes management outcomes in this population. This is crucial considering the role of institutional racism and implicit bias in health care settings and how these dynamics create racial disparities in health and health care outcomes.^13^

The following sections highlight four patient experience factors that have been investigated in the literature: patient-provider communication (PPC), patient-provider racial and ethnic concordance, patient-provider gender concordance, and satisfaction with health care provider.

### Patient-provider communication

PPC is identified as an important and modifiable factor associated with adherence to medication and lifestyle recommendations, diabetes self-management practices, and glucose control among adults with DM.^14–16^ High-quality PPC is characterized by shared decision making, active listening, respectful and compassionate care during clinical encounters.^17^ Racial differences in PPC among general adult patient populations have been documented for over 30 years.^18^ Racial and ethnic minority groups, including African American and Hispanic patients, are more likely to report experiencing lower quality PPC, a phenomenon that may contribute to racial health disparities.^19^ Relatively few studies have investigated racial differences in PPC among adults with DM.^20^ One study reported no racial and ethnic differences in PPC domains except for experiences of discrimination or disrespectful office staff.^21^

### Patient-provider racial and ethnic concordance

Patient-provider concordance, an important factor in patient experience, is often defined as a “shared identity between the health care provider and the patient based on a demographic attribute such as race and ethnicity or gender.”^22(p. 199)^ Research on the effects of patient-provider race and ethnicity concordance (defined as the patient and provider having the same racial or ethnic identity) on patient health outcomes is mixed.^23–25^

However, patient-provider race and ethnicity concordance has been extensively studied in general patient populations and is associated with higher levels of patient trust in their provider and perceived quality of care.^26,27^ Studies examining patient-provider race and ethnicity congruence are limited. One study reported that patient-provider race and ethnicity congruence among adults with DM was not associated with chronic disease management or treatment intensification.^28^

### Patient-provider gender concordance

Research on the effects of patient-provider gender concordance (defined as the patient and provider having the same gender identity) on general patient health outcomes is also mixed.^29,30^ In general, patient-provider gender concordance has been associated with increased patient trust in the provider and longer care visits.^31^ Less is known about patient-provider gender concordance among DM patients. One study reported that female DM patients with gender concordant providers demonstrated better glucose control.^30^

Overall, very little is known about the extent to which non-pregnant, women of childbearing age with DM have providers who have similar racial and ethnic and gender identities.

### Patient satisfaction

Patient satisfaction is an individual's subjective evaluation of their health care experience.^32^ It has become a key focus within health care institutions over the past decade.^33^ Patient satisfaction is often used by health care administrators to evaluate the success of the health care providers and to compare their institutions' performance with other health care institutions.^33^ Prior research has demonstrated that higher patient satisfaction is associated with less emergency department use, greater inpatient use, greater patient retention, and better patient-provider relationships.^34–36^

In general, several earlier studies have documented racial/ethnic differences in patient satisfaction with health care providers.^37–40^ Among patients with DM, however, less is known about their satisfaction with their health care providers. Available evidence suggests that patient satisfaction, among individuals with Type 2 diabetes, was associated with lower blood glucose levels, better general diet, and higher quality of life.^41^ Earlier research has reported low patient satisfaction among patients with DM.^42^

A more recent study reported that among patients with Type 2 diabetes, patient satisfaction was associated with lower blood glucose levels, better general diet, and higher quality of life.^41^ Earlier research has documented racial/ethnic differences in patient satisfaction among adults with DM in the U.S.^43^

To date, no identifiable studies of non-pregnant women of childbearing age have investigated racial differences in patient experience.

### Theoretical framework

The Interaction Model of Client Health Behavior (IMCHB)^44^ is a patient-centered model explaining the associations between the unique characteristics of an individual (patient singularity), the patient-provider interaction (interpersonal and communicative processes between patient and provider during clinical encounters), and intermediate patient outcomes (perception of care received, diabetes self-efficacy, and diabetes management). The modified IMCHB framework used to guide the present study is published elsewhere.^9^

This modified framework postulates that the *patient singularity* (*e.g.,* demographic characteristics of the patient population and perceived health status) can shape the *patient experience* (*e.g.,* communication, congruence satisfaction), *intermediate patient outcomes* (*e.g.,* ratings of care received, diabetes self-efficacy, and diabetes management), and *longer-term patient outcomes* (*e.g.,* glucose control, improved maternal health outcomes). It is likely that the factors related to patient characteristics, patient experience, and intermediate patient outcomes interact with each other. Also, the intermediate patient outcomes are believed to directly impact women's metabolic and endocrinological health and subsequently future maternal and infant health outcomes.

### Study objectives

The present study focuses on four patient experience variables (PPC, patient-provider race and ethnicity concordance, patient-provider gender concordance, and patient satisfaction with provider). More specifically, the purpose of this study is to identify potential racial and ethnic differences in patient experience among non-pregnant, women of childbearing age with DM. The authors hypothesized that there will be statistically significant differences in (1) ratings of PPC quality, (2) patient-provider race concordance, (3) patient-provider gender concordance, and (4) ratings of satisfaction with health care provider among Hispanic, NH Black or African American, and NH women of other or multiple races compared to NH White women of childbearing age with DM.

## Materials and Methods

### Sample

The sample was created using 7 years (2012–2018) of cross-sectional data from the public-use Medical Expenditure Panel Survey (MEPS) household consolidated files. The MEPS collects data on demographic characteristics, socioeconomic factors, individual health conditions (*e.g.,* diabetes diagnosis), and experiences with health care providers using a multi-stage panel design.^45^ Participants complete five rounds of interviews over a 2-year period consisting of household interviews and self-administered questionnaires.

The MEPS sample for each panel is a sub-sample of participants who completed the National Health Interview Survey from the previous year.^46^ Further details of the MEPS data collection methods, sampling, and complete questionnaires are available on the MEPS website.^47^ The sample was limited to non-pregnant (2012–2017 only) women of childbearing age (18–45 years) with DM. The MEPS did not collect data on current pregnancy status in 2018.^45^ The final size included 763 women representing 903,670 women when weighted.

### Measures

#### Independent variable

The independent variable in this study was race and ethnicity. Race and ethnicity were measured by asking participants “what is {your/person's} race?” and “{are/is}{you/person} Hispanic, Latino, or of Spanish origin?^45^” Participants were able to select one or more races, including White, Black or African American, Asian, American Indian or Alaska Native, and other races. Responses were collapsed to create a new variable comparing NH Whites, NH Blacks or African Americans, Hispanics, and NH adults who reported other or multiple races.

#### Dependent variables

The dependent variables of interest in this study were patient experiences, specifically PPC quality, patient-provider racial and ethnic concordance, patient-provider gender concordance, and satisfaction with health care provider. All dependent variables were collected during rounds two and four. PPC and satisfaction with health care provider variables were not collected in 2018.

##### Patient-provider communication

A combined measure of PPC quality was created using seven domains of PPC quality. Adults were asked to report how often in the past 12 months doctors or other health professionals “listen carefully to you,” “explain things in a way that was easy to understand,” “show respect for what you had to say,” and “spend enough time with you” on a Likert scale (1 = never, 2 = sometimes, 3 = usually, 4 = always). Adults were asked whether (yes or no) a doctor or other health professional would “give instructions about what to do about a specific illness or health condition.”

Adults who reported that their health care provider gave them instructions were also asked how often the health care provider asked them “to describe how you were going to follow these instructions” and “how often were these instructions easy to understand.” A dichotomous variable was created to compare providers who “always/yes” versus “not always/no” (usually, sometimes, or other) exhibited each domain of PPC quality. This method has been used in previous studies using MEPS public-use data to evaluate PPC quality.^48–50^

A combined measure was created to identify adults who reported that their health care providers “always” exhibited all domains of PPC quality versus those who did “not always” (usually, sometimes, never) demonstrate all domains of PPC quality based on previous studies using MEPS public-use data.^48,51,52^ Each domain of PPC quality was weighted equally.

##### Patient-provider racial/ethnic concordance

To determine patient-provider racial/ethnic concordance, the MEPS asks individuals who saw a health care provider in the past 12 months to specify “what is provider's race?” Individuals can specify whether their provider is White, Black or African American, Asian, Indian/Native American/Alaska Native, other Pacific Islander, or some other race. Individuals were also asked “is provider Hispanic or Latino?” Patient-provider racial/ethnic concordance was measured by comparing individual race and ethnicities with their provider.

For example, individuals who reported an NH White race with corresponding NH White provider, NH Black or African American race with corresponding Black provider, and Hispanic ethnicity with corresponding Hispanic provider were categorized as being patient-provider “racial/ethnic concordant.” Individuals who did not report having a provider with the same racial or ethnic background were categorized as patient-provider “racial/ethnic discordant.” This method has been used in previous research.^29^

##### Patient-provider gender concordance

To determine patient-provider gender concordance, the MEPS asks individuals to specify “is provider male or female?” Patient-provider gender concordance was measured by comparing individual genders with their provider. Female individuals who reported their provider was female and male individuals who reported their provider was male were categorized as being “patient-provider gender concordant.” Individuals who did not report having a provider with the same gender will be categorized as “patient-provider gender discordant.”

##### Satisfaction with health care provider

The MEPS includes a set of variables that are organized under a “satisfaction with the provider” category. These variables were designed to reflect the patient's confidence in, and satisfaction with, their health care provider.^46^ On the MEPS, individuals were asked to rank (1 = never, 2 = sometimes, 3 = usually, 4 = always), “if there were a choice between treatments, how often would a provider ask you to help make the decision?”

Individuals were also asked whether providers (yes or no) “usually ask about prescription medications and treatments other doctors may give them” and whether they “present and explain all options” to them. Further, individuals were asked: “thinking about the types of medical, traditional and alternative treatments that you are happy with, how often does a medical person/provider show respect for these treatments?” A combined variable was created to identify adults who were satisfied with their health care providers on all four aspects of care coordination compared with those who were less satisfied.

#### Covariates

Covariates that we evaluated included age, marital status (never married, married, or divorced/widowed/separated), education level (no degree/less than high school [HS], HS graduate or GED, bachelor's degree or higher), poverty (income <200% federal level, income >200% federal level), health insurance (any private, public only, none), and perceived health status (poor/fair, good/very good/excellent).

### Statistical analysis

Bivariate analyses (weighted chi square tests, *p* < 0.05) were conducted to make comparisons between racial and ethnic groups for all covariates and independent variables.

Crude and adjusted logistic regression models were used to determine associations between race/ethnicity and (1) PPC, (2) patient-provider race concordance, (3) patient-provider gender concordance, and (4) satisfaction with health care providers before and after adjusting for covariates. Data analysis was conducted using STATA 17.0 SVYSET procedures to account for the design features of the MEPS complex sample (StataCorp, College Station, TX).

Weights were divided by the total number of years included in the analysis based on the analytical guidance from MEPS (6 years for PPC and satisfaction with health care provider; 7 years for patient-provider race and gender concordance).^46^ *Post hoc* power estimates were not performed because there is an inappropriate approach for studies using secondary datasets and there are no identifiable comparative studies with the same population.^53^

This study using de-identified publicly available data did not meet the federal definition of human subjects' research (45 CFR 46); therefore, approval by our institutional review board was not required.

## Results

### Bivariate results

Differences in sample characteristics of women of reproductive age by racial and ethnic group are provided in Table 1. Although similar across groups (*p* = 0.6021, the mean age was highest among Hispanic women (37.2 years). Hispanic (55.8%), NH White (52.6%), and NH women of other or multiple races (46.8%) were more likely to be married than NH Black or African American women (*p* = 0.0047).

**Table 1.** Cross-Tabulations^a^ Between Race/Ethnicity, Selected Characteristics, and Patient Experience Outcome Variables, 2012–2018 Medical Expenditure Panel Survey, *n* = 763

	NH White *n = *239 *n* (%)^a^	NH Black/African American *n = *207 *n* (%)^a^	Hispanic *n = *241 *n* (%)^a^	NH other or multiple race *n = *76 *n* (%)^a^	*p*
Age, mean (SE)	36.3 (0.51)	36.8 (0.61)	37.2 (0.52)	36.1 (1.08)	0.6021
Marital status					0.0047
Never married	77 (31.1)	118 (53.2)	77 (30.9)	26 (32.2)	
Married	122 (52.6)	47 (27.5)	121 (55.8)	32 (46.8)	
Divorced/widowed/separated	40 (16.3)	42 (19.3)	43 (13.3)	18 (21.0)	
Education					<0.0001
No degree/less than HS	16 (5.3)	37 (22.5)	95 (40.0)	6 (7.2)	
HS graduate/GED	137 (63.7)	109 (55.1)	94 (47.8)	39 (55.8)	
Bachelor's degree or higher	60 (31.0)	31 (22.5)	22 (12.2)	26 (37.0)	
FPL					0.0330
<200% FPL	119 (40.6)	135 (53.5)	162 (55.2)	43 (55.0)	
≥200% FPL	120 (59.4)	72 (46.5)	79 (44.8)	33 (45.0)	
Health insurance					0.0007
Any private	142 (67.5)	96 (53.1)	75 (40.8)	37 (55.8)	
Public only	84 (27.3)	88 (36.1)	110 (39.6)	33 (35.6)	
Uninsured	13 (5.2)	23 (10.8)	56 (19.6)	6 (8.6)	
Perceived health status					0.1386
Poor/fair	61 (31.1)	37 (19.7)	41 (18.5)	17 (28.5)	
Good/very good/excellent	177 (68.9)	170 (80.3)	199 (81.5)	59 (71.5)	
PPC (*n* = 533)					0.9122
Not always	136 (82.4)	125 (81.1)	130 (83.0)	36 (78.8)	
Always	29 (17.6)	35 (18.9)	31 (17.0)	11 (21.2)	
Patient-provider race concordance (*n* = 410)					<0.0001
Patient-provider NOT same race	23 (22.4)	184 (87.4)	37 (63.6)	27 (73.6)	
Patient-provider same race	82 (77.6)	23 (12.6)	27 (36.4)	7 (26.4)	
Patient-provider gender concordance (*n* = 299)					0.3094
Patient-provider NOT same gender	60 (51.7)	43 (46.5)	41 (52.1)	24 (67.8)	
Patient-provider same gender	50 (48.3)	45 (53.5)	24 (47.9)	12 (32.2)	
Provider satisfaction (*n* = 439)					0.7002
Not satisfied	68 (53.7)	67 (50.1)	71 (52.5)	23 (60.8)	
Satisfied	60 (46.3)	65 (49.9)	67 (47.5)	18 (39.2)	

^a^ Unweighted frequencies, weighted percentages reported.

FPL, federal poverty level; GED, general education development; HS, high school; MEPS, Medical Expenditure Panel Survey; NH, non-Hispanic; PPC, patient-provider communication; SE, standard error.

Hispanic and NH Black or African American women were more likely to report having no degree or less than an HS level of education (40% and 22.5% respectively) than NH White and women of other or multiple races (*p* < 0.0001). NH White women were significantly more likely to report higher income >200% of the federal poverty level (59.4%) compared with all other racial and ethnic groups (*p* = 0.0330). Similar results were found for health insurance coverage.

NH White women were significantly more likely to report having any private health insurance (67.5%) compared with NH Black or African American (53.1%), Hispanic (40.8%), or NH women of other or multiple races (55.8%) (*p* = 0.0007). No statistically significant difference was found when perceived health status was measured across racial and ethnic groups (*p* = 0.1386).

Bivariate analyses also indicate no statistically significant differences in PPC quality (*p* = 0.9122). Among all racial and ethnic groups, the percent of women indicating their health care provider always provided PPC quality was low. No statistically significant difference was found when satisfaction with health care provider was measured across racial and ethnic groups (*p* = 0.7002), with less than half of women in each racial and ethnic group reporting satisfaction.

No statistically significant difference was found when patient-provider gender concordance was measured across racial and ethnic groups (*p* = 0.3094). A statistically significant difference was found when patient-provider racial/ethnic concordance was measured across racial and ethnic groups (*p* < 0.0001).

### Regression results

#### Patient-provider communication

Crude and adjusted regression results are provided in Table 2. There were no statistically significant differences in ratings of PPC quality among Hispanic, NH Black or African American, or NH women of other or multiple races in comparison to NH White women before or after adjusting for covariates.

**Table 2.** Crude and Adjusted Odds Ratios (95% Confidence Intervals) for Race/Ethnicity and Ratings of Patient-Provider Communication, Racial/Ethnic Concordance, Gender Concordance, and Patient-Provider Satisfaction Among Racial and Ethnic Groups, 2012–2018 Medical Expenditure Panel Survey

	Ratings of patient-provider communication		Patient-provider racial and ethnic concordance		Patient-provider gender concordance		Provider satisfaction	
	Crude	Adjusted^a^	Crude	Adjusted^a^	Crude	Adjusted^a^	Crude	Adjusted^a^
Race/ethnicity								
NH White (ref)	1.00	1.00	1.00	1.00	1.00	1.00	1.00	1.00
NH Black/African American	1.09 (0.56–2.10)	1.06 (0.55–2.04)	0.04 (0.02–0.10)	0.04 (0.01–0.11)	1.23 (0.57–2.67)	1.32 (0.57–3.07)	0.91 (0.48–1.71)	0.74 (0.33–1.68)
Hispanic	0.95 (0.49–1.86)	0.88 (0.42–1.85)	0.17 (0.07–0.38)	0.18 (0.06–0.50)	0.98 (0.44–2.19)	1.09 (0.42–2.85)	0.87 (0.49–1.53)	0.83 (0.40–1.72)
NH other or multiple race	1.26 (0.50–3.20)	0.87 (0.32–2.37)	0.10 (0.03–0.32)	0.12 (0.04–0.34)	0.51 (0.21–1.25)	0.49 (0.18–1.29)	0.65 (0.28–1.48)	0.55 (0.24–1.25)

^a^ Adjusted for age, marital status, education, poverty level, health insurance, and perceived health status.

#### Patient-provider racial and ethnic concordance

In crude models, NH Black or African American women had lower odds (OR = 0.04; 95% CI = 0.02–0.10) of seeing a health care provider of the same race compared with NH White women. Results remained consistent (aOR = 0.04; 95% CI = 0.01–0.11) after adjusting for age, marital status, education, poverty level, health insurance, and perceived health status. Similar results were found among Hispanic and NH women of other or multiple races.

In adjusted models, Hispanic women had lower odds (aOR = 0.18; 95% CI = 0.06–0.50) of seeing a health care provider of the same race/ethnicity compared with NH White women. Further, NH women of other or multiple races had lower odds (aOR = 0.12; 95% CI = 0.04–0.34) of seeing a health care provider of the same race/ethnicity compared with NH White women after adjusting for the aforementioned covariates.

#### Patient-provider gender concordance

There were no statistically significant differences in patient-provider gender concordance among Hispanic, NH Black or African American, or NH women of other or multiple races in comparison to NH White women before or after adjusting for covariates.

#### Satisfaction with health care provider

There were no statistically significant differences in ratings of satisfaction with their health care provider among Hispanic, NH Black or African American, or NH women of other or multiple races in comparison to NH White women before or after adjusting for covariates.

## Discussion

This study examined racial and ethnic differences in patient experiences of diabetes care among non-pregnant women of childbearing age in the U.S., focusing on (1) PPC quality, (2) patient-provider racial and ethnic concordance, (3) patient-provider gender concordance, and (4) patient satisfaction with health care provider. This is the first known study to examine racial and ethnic differences in patient experiences of nonpregnant women of childbearing age with DM and contributes to the dearth of literature related to this patient population.

This study reports no statistically significant differences by race or ethnicity for (1) ratings of PPC quality, (2) patient-provider gender concordance, or (3) ratings of satisfaction with their health care provider. The study data indicate that participants, regardless of race/ethnicity, reported low PPC quality and dissatisfaction with their health care provider. Although the present study did not make comparisons to men, our findings have some relevance to prior research suggesting that, compared with their male counterparts, women with DM are less likely to be satisfied with their diabetes care^54^ and are less likely to receive the recommended diabetes care from health care providers.^55,56^

In fact, health care providers have cited lack of time and knowledge as barriers to providing the recommended care to reproductive age women with diabetes.^57^ It is difficult to compare the results of the present study with the extant literature on general patient populations, because the findings are inconsistent. For example, although some studies have reported no racial and ethnic differences in PPC quality in general patient populations,^21^ others have reported racial and ethnic differences in patient satisfaction among adults with DM.^43^ Therefore, more research is needed to understand the patient experiences of racially and ethnically diverse nonpregnant women of childbearing age with DM and how unique racial and gender disparities may manifest.

There were no statistically significant differences in patient-provider gender concordance by race/ethnicity. Among racial/ethnic subgroups, with the exception of participants in the NH other or multiple race category, about half of the participants did not have a provider of the same gender. There were statistically significant racial/ethnic differences for patient-provider racial and ethnic concordance, with NH Black, Hispanic, and NH other/multiple race women being less likely than NH White women to have a provider of the same race/ethnicity. Although these findings are not surprising, they are concerning considering the existing gender disparities, which are compounded by racial/ethnic disparities, in diabetes care quality, care satisfaction, and self-management.^7,54–56^

In general, research shows mixed findings when examining patient-practitioner racial and ethnic concordance, with many studies citing small sample sizes as a limitation in determining associations and effect sizes.^24,58^ One reason for this is the lack of practitioner representation for NH Black and Hispanic providers.^59^ It is possible that racial and ethnic representation has increased slightly over the past decade, enough for findings to have adequate data to reach clinical significance.

Research also shows an association between racial and ethnic concordance and the likelihood of patients visiting their provider^60^ and better communication across many patient-physician communication domains.^23^ Altogether, since patient-provider racial concordance can improve health outcomes,^61^ and the population in the U.S. is an increasingly majority-minority population,^62^ it is of the utmost importance to encourage better health care practitioner representation that matches the population served. It is important to emphasize, however, that although increased health care practitioner representation is important, it is up to all health care providers regardless of their racial/ethnic identity to provide optimal care to all patients.

As illustrated in the IMCHB framework,^9,44^ the ways in which patient singularity (*i.e.,* characteristics of the patient population) and intermediate factors (*i.e.,* ratings of care received, diabetes self-efficacy, and diabetes management) interact are vital for long-term health outcomes (*i.e.,* glucose control, improved maternal health outcomes) to reduce risks of adverse maternal and infant health outcomes. Demographic characteristics such as race and/or ethnicity are of particular importance. For example, newborn-physician racial concordance was associated with a significant reduction in mortality for Black infants.^61^

Since women with DM are often at higher risk for maternal and infant complications than women without DM, it is important to continue investigating the processes to mitigate negative health outcomes for both.^63^ Yet, it is likely that other factors outside the IMCHB may influence these outcomes even while examining specific demographic variables. For example, a recent study showed that Black concordant patient-provider dyads report higher levels of similarity than other dyads, with provider self-disclosure leading to higher levels of trust, rapport, similarity, likeability, intention to disclose, satisfaction, behavioral intention to continue using the provider, and intention to recommend the provider.^64^

Therefore, it is important for health care providers to acknowledge the impacts their own self-disclosure could have for patient satisfaction and patient health outcomes and receive training on how to manage self-disclosure in an ethical and professional manner.

The present study has several limitations. First, the data were collected from cross-sectional surveys, limiting causal inference. For future studies, data from longitudinal studies and randomized control trials should be included to mitigate this limitation. Second, MEPS is based on self-report, which may bias the results as patient self-reports of communication quality may differ from objective measures (*e.g.,* patient-provider observations). To limit this challenge, reports, data and case histories from physicians and patients can be collected along with participant self-reports to improve validity. Third, there may be other factors not measured in this study that explain why there were no statistically significant racial or ethnic differences in PPC quality, gender concordance, and ratings of satisfaction with provider. In the future, studies should focus on determining a potential measuring variable to find the differences and challenges in PPC among racial and ethnic minority groups. Fourth, there was no differentiation between type 1 and type 2 diabetes within the MEPS, with participant groups classified based on the presence or absence of diabetes. Inclusion of type of diabetes can give a better idea on PPC and satisfaction depending on different patient needs. Future studies should focus on making the differentiation between these two types of diabetes.

Overall, the study findings suggest a need for patient-centered intervention strategies that can improve PPC quality and patient satisfaction among nonpregnant women of childbearing age with diabetes regardless of race/ethnicity. The study findings may have implications for health policy and practice.

First, as research shows that patients with racial and ethnic concordance with their health care providers have increased likelihood of visiting their provider,^60^ it is important to engage and recruit diverse health care practitioners. Further, it is also vital for health care facilities and systems to retain these practitioners that can be achieved through mentorship practices for people of historically marginalized backgrounds (due to race, ethnicity, gender, or the intersectionality of these and other characteristics) in clinical settings. Until this is accomplished, it is of the utmost importance for health care delivery to be provided using gender-specific and culturally appropriate and tailored approaches, particularly for racial and ethnic discordant patient-provider relationships. Second, it is important to assess the current measures ascertaining individuals' perceptions of PPC, as the established measures are typically tested and validated in predominantly White contexts.^65,66^

Creating and testing culturally appropriate and tailored measures among racially and ethnically diverse women with DM may increase understanding as to the relationships between patient-provider racial and ethnic concordance and other variables, such as ratings of satisfaction with provider, that cannot be accurately assessed without a more nuanced approach.

## Conclusion

Our findings contribute to the dearth of literature related to nonpregnant women of childbearing age with DM and identify racial and ethnic differences in patient experiences among non-pregnant women of childbearing age with DM in the U.S. DM is an increasingly common condition, with associated health care costs growing at alarming rates.

There is a need for more research focused on women with DM, which can inform and improve health care delivery strategies that impact nonpregnant women of childbearing age. These findings can inform interventions toward improving PPC, all with the intent of improving health outcomes for women with DM across the U.S.

## Data Availability

The data reported in the final article are publicly available (https://meps.ahrq.gov/mepsweb/).

## Abbreviations Used

ADA: American Diabetes Association
aOR: adjusted odds ratio
CI: confidence interval
DM: diabetes mellitus
FPL: federal poverty level
GED: general education development
HRSA: Health Resources and Services Administration
HS: high school
IMCHB: Interaction Model of Client Health Behavior
MEPS: Medical Expenditure Panel Survey
NH: non-Hispanic
PPC: patient-provider communication
SDAR: Secondary Data Analysis Research
SE: standard error
U.S.: United States
